# PReS-FINAL-2245: A rare case of subcutaneous panniculitis-like T cell lymphoma in a 9-month-old child

**DOI:** 10.1186/1546-0096-11-S2-P235

**Published:** 2013-12-05

**Authors:** A Kozlova, D Abramov Dmitriy, N Miakova, A Maschan, A Shcherbina

**Affiliations:** 1 Immunology, Federal Research and Clinical Center of Pediatric Rheumatology, Oncology and Immunology, Moscow, Russian Federation

## Introduction

Panniculitis is an inflammatory disorder of subcutaneous adipose tissue, frequently accompanied by vaculitis and systemic involvement. The peak age of patients is 20-30 years, and it is fairly rare in children, especially in infants.

Therefore, panniculitis-like lesions in young children require differential diagnostic procedures and sometimes joined efforts of rheumatologists, hematologists and oncologists.

## Objectives

to illustrate the difficulties in the diagnosis of subcutaneous panniculitis-like T cell lymphoma in an infant.

## Methods

A 9-month-old child was admitted to our clinic in September 2012 for treatment of his panniculitis. First lesion on the face appeared in June 2012, abscess was suspected and it was surgically drained. Yet, in July 2012 there were new lesions and the boy started having daily spikes of fever. He was started on antibiotics without effect, the number and the size of lesions continued to grow. The biopsy was performed and based on the pathology results and symptoms the diagnosis of Weber panniculitis was made and the boy was referred to our center.

## Results

At the initial examination the boy appeared not well, very pale, had daily fevers, and multiple edematous purple skin lesions on his face, legs, arms. He had large peripheral lymph nodes (up to 3 cm), hepatosplenomegaly, anemia (hemoglobin to 88 g/L), mild leucopenia (WBC 3.2 × 10^9^/ml), increased transaminases and lactate dehydrogenase. Chest X-ray, ultrasound of the abdomen, chest and abdomen CT were unremarkable, except for hepatosplenomegaly and obviously enlarged peripheral lymph nodes. Considering the age of the patient the diagnosis of panniculitis seemed questionable and the biopsy was repeated. As a result panniculitis-like T-cell lymphoma of the skin was diagnosed, the child was started on CHOP protocol treatment with dramatic improvement of his general condition and reduction of the lesions.

## Conclusion

The case demonstrates the importance of critical approach to rheumatological patients that don't fit the general understanding of the pathology and epidemiology of the disorder.

## Disclosure of interest

None declared.

